# Supplementary material for: Maternal Fat-1 Transgene Protects Offspring from Excess Weight Gain, Oxidative Stress, and Reduced Fatty Acid Oxidation in Response to High-Fat Diet
Source: Nutrients. 2020 Mar 14;12(3):767. doi: 10.3390/nu12030767 (PMC7146584; doi:10.3390/nu12030767)
Supplement: Supplementary file 1 [file nutrients-12-00767-s001.pdf]

Article

Maternal omega-3 fatty acid profile protects offspring from excess weight gain, oxidative stress, and reduced fatty acid oxidation in response to high-fat diet

Boyle and Magill-Collins et al.

Supplementary Tables & Figure

Table S1. Composition of diets

	Control Diet<br>Research Diets<br>D12450B	High-fat Diet<br>Research Diets<br>D12451
Energy Density	3.8 kcal/g	4.7 kcal/g
Total Protein	20 %	20 %
Total Carbohydrate	70 %	35 %
Total Fat	10 %	45 %
Fat composition	lard, soybean oil	lard, soybean oil
Saturated Fats	24 %	32 %
Monounsaturated Fats	30 %	36 %
Polyunsaturated Fats	47 %	33 %
Trans Fats	0	0
n-3 fatty acids	1.7 g/100 g	6.6 g/100 g
n-6 fatty acids	0.2 g/100 g	0.5 g/100 g
n-6:n-3 ratio	8.4	13.1

Table S2. Antibodies Used

Antibody	Manufacturer	Cat. No.	Ab Dilution
GAPDH	Cell Signaling Technology	4970	1:50
MnSOD	Enzo Life Sciences	ADI-SOD-110	1:1,000
SIRT3	Cell Signaling Technology	2627	1:1,000

Figure S1. Skeletal Muscle Citrate Synthase Activity

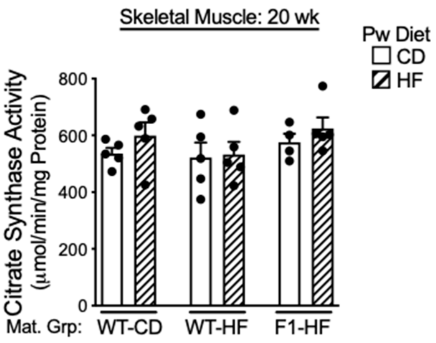
Skeletal Muscle: 20 wk
Pw Diet
CD
HF
Citrate Synthase Activity
(µmol/min/mg Protein)
800
600
400
200
0
Mat. Grp:
WT-CD
WT-HF
F1-HF
